# Intussusception: Some Remarks on Its Diagnosis and Treatment

**Published:** 1908-09-12

**Authors:** 


### SURGERY.

### INTUSSUSCEPTION: SOME REMARKS ON ITS DIAGNOSIS AND TREATMENT

The immediate recognition of the presence of an intussusception in an infant is a matter of the greatest possible importance, for this condition may well be included in the category of urgent surgical emergencies, and any delay in realising the gravity of the situation may endanger the life of the child.

The diagnosis is more or less obvious in some cases, but in others often presents great difficulties. This is partly due to the fact that the condition commonly occurs in children between the ages of three months and three years; and even if the child is of an age to talk at all, the information which can be obtained by questioning him is not likely to be of any great value. The testimony of the mother, if she be an observant woman, is generally much more helpful. The onset is in almost all cases sudden, and this is denoted by the fact that the child, which was previously in its ordinary state of health, becomes all at once abnormally quiet, and may become blanched in the face. He often lies on his back with his legs drawn up, and if the abdominal pain is severe, as it usually is, he will demonstrate this by holding his hands to his abdomen. A young child may also scream at the beginning of the attack.

There is often diarrhœa before, and vomiting at, the beginning of the attack; but as both these phenomena are associated with the majority of the ailments of children, they do not in themselves constitute a factor of any diagnostic importance. Besides, curious though it may seem in a condition which is to all intents and purposes one of acute intestinal obstruction, vomiting may be absent throughout, and the writer's experience of these cases has led him not to attach too much importance to the presence or absence of this symptom. After the onset the abdominal pain is persistent, but is subject to exacerbations, which cause the child to writhe and scream. These exacerbations are easily intelligible; they are caused by the peristaltic efforts of the intestine to drive on the intussusception. For an intussusception is regarded by the intestine in the same light as a foreign body or a mass of retained fæcal matter, and an attempt is accordingly made to promote its passage. At any rate the condition is probably started in the first place by the presence of a thickening in the bowel wall, possibly of an inflammatory nature—that is to say, by a localised enteritis. If this explanation is accepted, it may account for the great relative frequency of the ileo-cæcal variety, in which the intussusception begins at the ileo-cæcal valve, for this is the narrowest portion of the intestine in children, and is thus especially liable to irritation. It may also account for the fact that after the reduction of an intussusception no apparent cause can be found to account for it, since a thickening which can be appreciated by the intestine itself may be so slight as to be impalpable to the hand of the surgeon. Rarely one comes across cases in which the intussusception can be definitely attributed to an attempt on the part of the bowel to drive on an intestinal polypus, but these are few and far between. The antecedent diarrhœa, already mentioned as frequently occurring, may also be regarded as evidence of the presence of an enteritis. The other conditions usually quoted in text-books of surgery as predisposing or exciting causes of intussusception, as, for instance, "congenital laxity of the mesentery" and "inco-ordination of peristalsis," appear to the writer to be vague and unconvincing phrases, carefully worded to conceal our ignorance; since, if they are critically examined, it will be seen that they cannot rest on a basis of observed fact, on which all clinical deductions should be founded.

To return, however, to the clinical course of the case, intestinal obstruction is generally absolute, and no fæces are passed; although, like all rules of surgery, this is not absolute, since the fæcal matter, which was in the colon at the time the intussusception started, may be discharged after a few hours. Sooner or later some blood and mucus is passed by the rectum, generally within twelve hours from the onset of the symptoms. This is a sign of the utmost importance, and may be regarded as almost pathognomonic of intussusception.

Satisfactory examination of the child is often a matter of the greatest difficulty, and in an ordinary case abdominal palpation may give no assistance, because the child resists vigorously and holds its abdominal muscles in a state of rigidity. Whenever, therefore, the presence of an intussusception is suspected, it is wise to administer an anæsthetic. This is a piece of advice which is the outcome of practical experience, for the writer has known several doubtful cases cleared up completely by this simple manœuvre. It is quite easy to give a child sufficient chloroform to render it quiet, and only a light anæsthesia is required to render the abdomen soft enough for the purposes of examination.

As soon as the child is quiet a systematic examination of the abdomen should be made. One is said to be able to find a "sausage-shaped" tumour. The epithet has been so constantly applied to this condition that it has now become classical. It is true that the tumour is often elongated, but anyone who expects to find such a swelling in all cases is doomed to many disappointments. On account of the great relative frequency of the ileo-cæcal variety it is well to start examining the abdomen in the region of the cæcum, and to follow the course of the large intestine. If this is done one is often able to recognise the existence of "Dance's sign," which consists of an absence of the normal resistance in the right iliac fossa, caused by the removal of the ileo-cæcal valve and cæcum from their normal position. The commonest place in which the tumour caused by the intussusception is found is in the right hypochondrium, where it lurks under the margin of the right lobe of the liver. But it must be remembered that it is astonishingly mobile, so that it may be more or less evident at one moment and apparently completely gone the next. More rarely it may be found in the left iliac fossa. In the rare condition in which one coil of small intestine is driven into the next one, the tumour may be found anywhere.

After complete examination of the abdomen the rectum should be digitally explored. In some cases no blood or mucus has been actually passed by the rectum, but is present and has been retained. In a small child rectal examination should be made with the little finger, and blood and mucus will almost always be found on it on withdrawal. The presence of blood and mucus in the rectum is easily explained. As the intussusception travels onwards and becomes larger, the mesentery becomes constricted and the circulation in the affected loop of intestine is arrested. So that besides obstruction to the contents of the gut there is obstruction to its blood supply; in other words, a genuine strangulation of the intestine results. This leads to œdema of the intussusception with consequent hæmorrhage into the lumen of the bowel, and it is the extravasated blood, combined with intestinal mucus, which is found in the rectum.

It may be laid down as an absolute rule that as soon as the presence of an intussusception has been diagnosed, the abdomen should be opened at the earliest possible moment, because a delay of even an hour is sufficient to impair the child's chances of recovery. In fact, the prognosis in any given case depends almost entirely on the number of hours which have elapsed since the onset of symptoms. The mortality in cases operated on within the first twenty-four hours is, roughly, 10 per cent., whereas if the operation is not performed until the third day the outlook is practically hopeless.

Attempts to reduce the intussusception by means of enemata should not be countenanced. This was the method employed before the practice of opening the abdomen in all cases was universally accepted. The child was laid on its back with the buttocks raised and an enema containing warm normal saline solution and brandy was administered by means of a tube and funnel, the latter being raised to a height of about two feet, the idea being to reduce the intussusception by mechanical pressure. It was said to be successful in early cases, some advocates of the method having recorded as many as 40 per cent. of successes in cases in which the enema was administered within twelve hours of the onset of symptoms. But it is at any rate certain that it must be exceedingly difficult to know whether reduction has been effected in this way or not; and, if the method did not fail completely, it often resulted in partial reduction only, which was as useless as complete failure, added to which there is a definite risk of damaging or even of bursting the intestine.

Immediate laparotomy is the only sane treatment. The abdomen should be opened by a vertical incision through the right rectus muscle. It is better to make the incision on the right side, because the intussusception is more usually found on that side on account of the relative frequency of the ileo-cæcal variety. As soon as the peritoneum is opened the tumour should be sought for, and when found reduction should be attempted by withdrawing the intussuscipiens from the intussusceptum, and not by pulling on the intussusceptum itself. In an early case reduction is generally effected readily, but after a few hours peritoneal adhesions form between the serous coat of the intussuscipiens and that of the intussusceptum, and this will render reduction doubly difficult. If, happily, this is successfully achieved, the intestines are returned to the abdomen and the wound sewn up. The shock of such an operation to a small child is, of course, very great, and no time must be lost unnecessarily. But if the child's general condition will stand it, it is well to spend a few extra minutes in sewing up the abdomen in layers; for this reason, that one of the commonest complications in the after treatment of these cases is that the sutures give way and the intestines are extruded through the wound. Apart from the fact that there seems to be a greater intra-abdominal tension in children than there is in adults, there is no obvious reason why this accident should occur more frequently after intussusception than it does after other abdominal operations. The treatment is comparatively simple. The child should be at once anæsthetised, the intestines irrigated with saline solution and replaced, and the wound sewn up as carefully as possible.

So far it has been supposed that the intussusception can be reduced; but if, when the abdomen has been opened, the intussuscepted portion is found to be gangrenous and reduction impossible, the prognosis is almost hopeless. The alternatives which lie before the surgeon are (1) to resect the gangrenous portion; (2) to make an artificial anus; or (3) to perform a lateral anastomosis above and below the intussusception and bring out the gangrenous portion into the wound and allow it to slough off. Whichever of these methods is preferred, the outlook is particularly unpromising, for on the one hand the child's condition is probably not good enough to stand a long operation as resection of the intussusception with end to end anastomosis is bound to be, and on the other a child with an artificial anus rarely survives the operation for any length of time, especially if, as in this case, the artificial anus is made in the small intestine. The late Mr. Greig Smith, of Bristol, suggested an ingenious operation; he advocated making a vertical incision in the intussuscipiens and removing the gangrenous intussusceptum through this, the result being similar to the condition left after Maunsell's operation for end to end anastomosis, but the writer has never seen it performed.

